# Imprint of 5-azacytidine on the natural killer cell repertoire during systemic treatment for high-risk myelodysplastic syndrome

**DOI:** 10.18632/oncotarget.6213

**Published:** 2015-10-21

**Authors:** Ebba Sohlberg, Aline Pfefferle, Sandra Andersson, Bettina C. Baumann, Eva Hellström-Lindberg, Karl-Johan Malmberg

**Affiliations:** ^1^ Department of Medicine, Center for Infectious Medicine, Karolinska Institutet, Karolinska University Hospital Huddinge, Stockholm, Sweden; ^2^ Department of Medicine, Center for Hematology and Regenerative Medicine, Karolinska Institutet, Karolinska University Hospital Huddinge, Stockholm, Sweden; ^3^ Department of Cancer Immunology, Institute for Cancer Research, Oslo University Hospital Radiumhospitalet, Oslo, Norway; ^4^ The KG Jebsen Center for Cancer Immunotherapy, Institute of Clinical Medicine, University of Oslo, Oslo, Norway

**Keywords:** myelodysplastic syndrome, natural killer cell, killer cell immunoglobulin-like receptors, 5-azacytidine, Immunology and Microbiology Section, Immune response, Immunity

## Abstract

5-azacytidine (5-aza) is a hypomethylating agent approved for the treatment of high-risk myelodysplastic syndrome (MDS). It is assumed to act by demethylating tumor suppressor genes and via direct cytotoxic effects on malignant cells. *In vitro* treatment with hypomethylating agents has profound effects on the expression of killer-cell immunoglobulin-like (KIR) receptors on natural killer (NK) cells, as these receptors are epigenetically regulated via methylation of the promoters. Here we investigated the influence of 5-aza on the NK-cell repertoire during cytokine-induced proliferation *in vitro* and homeostatic proliferation *in vivo* in patients with high-risk MDS. *In vitro* treatment of NK cells from both healthy donors and MDS patients with low doses of 5-aza led to a significant increase in expression of multiple KIRs, but only in cells that had undergone several rounds of cell division. Proliferating 5-aza exposed NK cells exhibited increased IFN-γ production and degranulation towards tumor target cells. MDS patients had lower proportions of educated KIR-expressing NK cells than healthy controls but after systemic treatment with 5-aza, an increased proportion of Ki-67^+^ NK cells expressed multiple KIRs suggesting uptake of 5-aza in cycling cells *in vivo*. Hence, these results suggest that systemic treatment with 5-aza may shape the NK cell repertoire, in particular during homeostatic proliferation, thereby boosting NK cell-mediated recognition of malignant cells.

## INTRODUCTION

Epigenetic modifications are a common hallmark of all human cancers [1] and can include alterations in methylation patterns where methyl groups are added or removed from CpG sites in DNA. In hematopoietic malignancies, the promoter CpG islands of tumor-suppressor genes are often hypermethylated and thus epigenetically silenced [2]. Myelodysplastic syndrome (MDS) is a group of clonal stem cell disorders characterized by ineffective hematopoiesis, a potential for progression to acute myeloid leukemia (AML) and which is connected to deregulation of innate immune and inflammatory signaling [3]. Abnormal cellular differentiation in MDS can frequently be linked to aberrant genome-wide and loci-specific DNA hypermethylation as well as mutations in genes that regulate epigenetic programs [4].

Several epigenetic drugs have been developed for cancer treatment, including the cytosine analogs 5-azacytidine (5-aza) and 5-aza-2′-deoxycytidine (decitabine). 5-aza has been shown to induce a clinical response in around 50% of patients with MDS and treatment is associated with prolonged survival [5]. For the last 10 years, 5-aza has been the first-line treatment for patients with high-risk MDS and acute myeloid leukemia (AML) with dysplastic features and 20-29% marrow blasts [6]. 5-aza and decitabine lack methylation sites thus effectively acting as DNA methyltransferase inhibitors (DNMTs). 5-aza particularly inhibits DNMT1, which is completely depleted after 5-aza exposure due to the formation of an irreversible covalent bond, leading to loss of DNA methylation marks [7, 8]. In this way, methylation-dependent silencing in cancer cell lines is reversed in a dose-dependent manner, where only low doses that do not disturb replication show hypomethylating effects [9].

Besides demethylation, administration of 5-aza may also delay the progression of MDS by influencing the immunological control of the malignant clone [10]. Natural killer (NK) cells express a vast array of activating receptors that sense cellular stress, including NKG2D, DNAM-1 and natural cytotoxicity receptors (NCRs) [11]. Stressed and transformed cells upregulate MHC class I chain related proteins A and B (MICA/B) and UL16-binding proteins (ULBPs) (NKG2DL) that interact with NKG2D, one of the major NK-cell activating receptors involved in tumor cell recognition [12]. Absence of transcription of NKG2DL, due to high levels of DNA methylation, can be detected in tumor cell lines but expression can be restored by treatment with demethylating agents [13, 14].

In addition to synergistic signaling through activating receptors, NK cell cytotoxicity is tightly controlled by the expression of MHC class I binding receptors including CD94/NKG2A and killer cell immunoglobulin-like receptors (KIRs). Variegated expression of these receptors in the NK cell repertoire confers tolerance to normal cells but also contributes to determine the functional potential of the cells in a process termed education [15, 16]. Interactions between inhibitory KIRs and/or CD94/NKG2A and their cognate HLA class I ligands at steady state influence the ability of the cell to respond to environmental changes, including the recognition of cells with low expression of HLA class I [17, 18]. KIRs are acquired late during NK cell differentiation and are also commonly expressed on terminally differentiated CD56^+^ T cells [19]. In both T and NK cells, KIR expression is regulated at the transcriptional level by epigenetic changes in a complex and synchronized fashion determined by the activities from multiple promoters [20]. KIR genes are consistently demethylated in cells expressing KIRs and methylated in cells without KIR expression [21, 22]. The transcriptional regulation of KIR genes leads to a stochastic expression of KIR proteins at the cell surface, where some NK cells do not express a single KIR while others express combinations of multiple KIRs [23, 24]. *In vitro*, NK cell lines and primary *ex vivo* expanded NK cells upregulate KIRs on their cell surface during decitabine [21, 22] and 5-aza stimulation [25].

In spite of existing *in vitro* data on the effects of hypomethylating agents on the NK cell compartment, little is known regarding the possible effects of 5-aza on NK cells *in vivo*. Given the high turn-over of NK cells in peripheral blood [26] we hypothesized that incorporation of 5-aza during NK cell homeostasis might lead to altered expression of KIR, thereby affecting their functionality through education and possibly impact their ability to mediate anti-tumor immunity. Moreover, we reasoned that changes in KIR expression might serve as a sensitive tool to monitor uptake of 5-aza into circulating cells *in vivo*, the evidence for which is sparse [27]. Our data show that expression of multiple KIRs was rapidly induced on NK cells during *in vitro* culture with physiologically relevant low doses of 5-aza. This effect was tightly linked to IL-2 driven cellular proliferation and therefore most prominent in less differentiated cells with high proliferative capacity. Longitudinal assessment of NK cells in MDS patients undergoing systemic 5-aza treatment revealed increased frequencies of KIR expression in Ki-67^+^ NK cells, indicative of 5-aza uptake during cell division *in vivo*. Interestingly, cycling NK cells exposed to 5-aza *in vitro* had higher degranulation and IFN-γ production in response to K562 target cells suggesting enhanced function post-5-aza exposure. Our data reveal an imprint of 5-aza on NK cells *in vivo* and support the notion that the therapeutic effects of 5-aza may be partially mediated via epigenetic remodeling of the immune system.

## RESULTS

### 5-aza increases KIR expression on proliferating NK cells *in vitro*

**Figure 1:**
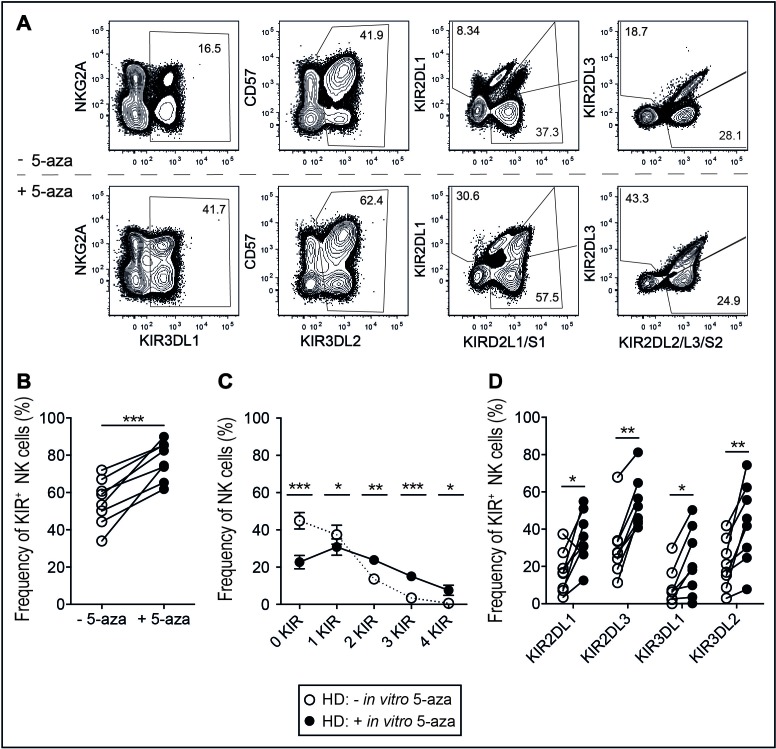
KIR repertoires in the NK cell population after *in vitro* 5-aza addition NK cells were isolated from healthy donor PBMC and cultured in 500U/ml of IL-2 for six days with or without the addition of 5-aza for the first four consecutive days. **A.** Gating scheme to identify subsets of CD56^+^ NK cells expressing single KIR and combinations thereof. Doublet cells were excluded based on an FSC-area versus FSC-height gate. Gates were set on live CD3^−^ cells as determined by staining with a dead cell marker (DCM) and anti-CD3. Shown in **B.** frequency of KIR^+^CD56^+^ NK cells, **C.** the number of expressed inhibitory KIRs and in **D.** each investigated KIR. HD *n* = 8.

To assess the effect of the hypomethylating drug 5-aza on the NK cell KIR repertoire of healthy individuals, NK cells were isolated from PBMC and cultured *in vitro* with IL-2 in the presence or absence of 5-aza. 5-aza was added consecutively to the culture at dose-levels in the range of those observed in plasma of patients receiving systemic treatment [28]. After six days the frequency of cells expressing KIRs was analyzed using a flow-cytometry panel that enabled identification of cells expressing single KIRs or combination thereof (Figure 1A). Addition of 5-aza significantly increased the frequency of total KIR-expressing NK cells, of NK cells co-expressing 2, 3 or 4 KIRs and of each of the analyzed inhibitory KIRs (Figure 1B-1D). In the three donors with group B KIR haplotype, a similar increase in the expression of KIR2DS1 was noted (as illustrated by one donor in Figure 1A).

**Figure 2:**
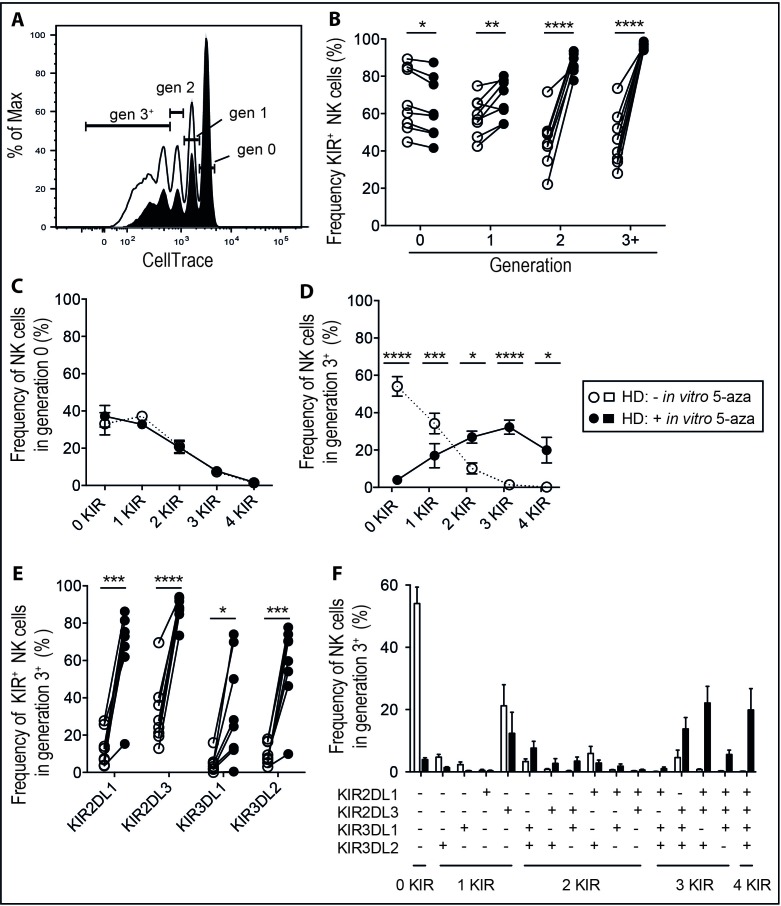
5-aza induces expression of multiple KIRs on proliferating NK cells NK cells were isolated from healthy donor PBMC and cultured in 500U/ml of IL-2 for six days with or without the addition of 5-aza for the first four consecutive days. Shown in **A.** CellTrace dilution of NK cells from one representative donor, open histogram represents cultures without 5-aza and black histogram with 5-aza. In **B.** the frequency of KIR^+^CD56^+^ NK cells in each generation, in **C.** the number of expressed KIRs in non-dividing NK cells or **D.** in those that had undergone three or more cell divisions (generation 3^+^). In **E.** the frequency of each investigated KIR in NK cells in generation 3^+^ and in **F.** resolution of their KIR repertoires. HD *n* = 8.

As the hypomethylating effects of 5-aza require incorporation into DNA during cell division [29], we stratified the analysis based on the number of cell divisions (Figure 2A) induced by IL-2. The effect of 5-aza on KIR expression was most evident in cycling cells, where nearly 100% of the cells expressed at least one KIR following three or more cell divisions (Figure 2B). This was in sharp contrast to cultures without 5-aza where we observed a gradual decline in KIR expression, presumingly due to the preferential proliferation of less differentiated KIR^−^ NK cells [30]. Notably, late generation NK cells also co-expressed multiple KIRs, which was rarely seen in non-dividing cells (Figure 2C-2E). To assess if 5-aza preferentially induced specific combinations of KIRs, we resolved the KIR repertoire of NK cells in generation 3^+^. Again, the frequency of NK cells expressing three or more KIRs was higher with addition of 5-aza, although no specific pattern in the KIR repertoire was noticed (Figure 2F).

### 5-aza-induced KIR expression is most evident in NKG2A^+^CD57^−^ NK cells

**Figure 3:**
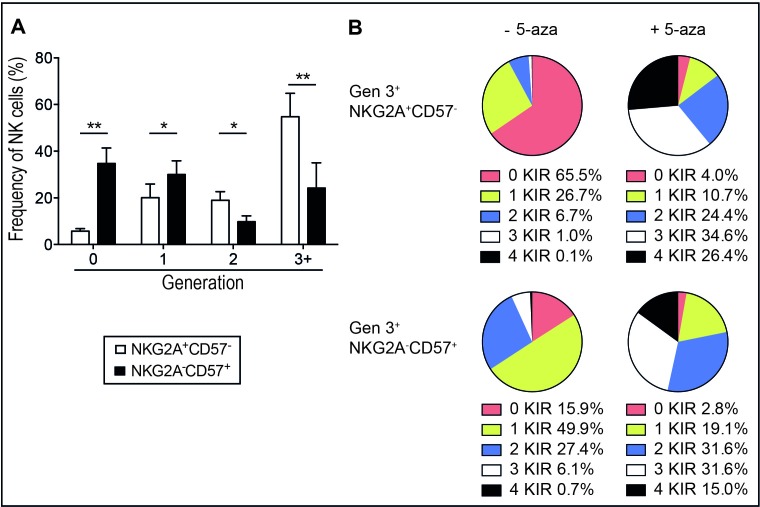
5-aza induced KIR expression on proliferating immature and mature NK cells NK cells were isolated from healthy donor PBMC and cultured in 500U/ml of IL-2 for six days with or without the addition of 5-aza for the first four consecutive days. In **A.** the frequency of immature NKG2A^+^CD57^−^ and mature NKG2A^−^CD57^+^ NK cells within each generation. In **B.** the mean frequencies of generation 3^+^ NKG2A^+^CD57^−^ and NKG2A^−^CD57^+^ NK cell subsets expressing 0-4 KIRs. HD *n* = 8.

To further explore the effect of 5-aza on NK cells, we stratified the analysis based on the stage of NK cell differentiation as determined by the expression of NKG2A and CD57. In agreement with previous results [30] less differentiated NKG2A^+^CD57^−^ NK cells proliferated the most in response to IL-2 (Figure 3A). Although 5-aza induced a significant increase in the expression of multiple KIRs in cycling cells of both subsets, the difference was most evident on NKG2A^+^CD57^−^ NK cells that had lower initial KIR expression and higher proliferation rates (Figure 3B).

### KIR repertoires of proliferating NK cells of MDS patients after *in vivo* 5-aza treatment

**Figure 4:**
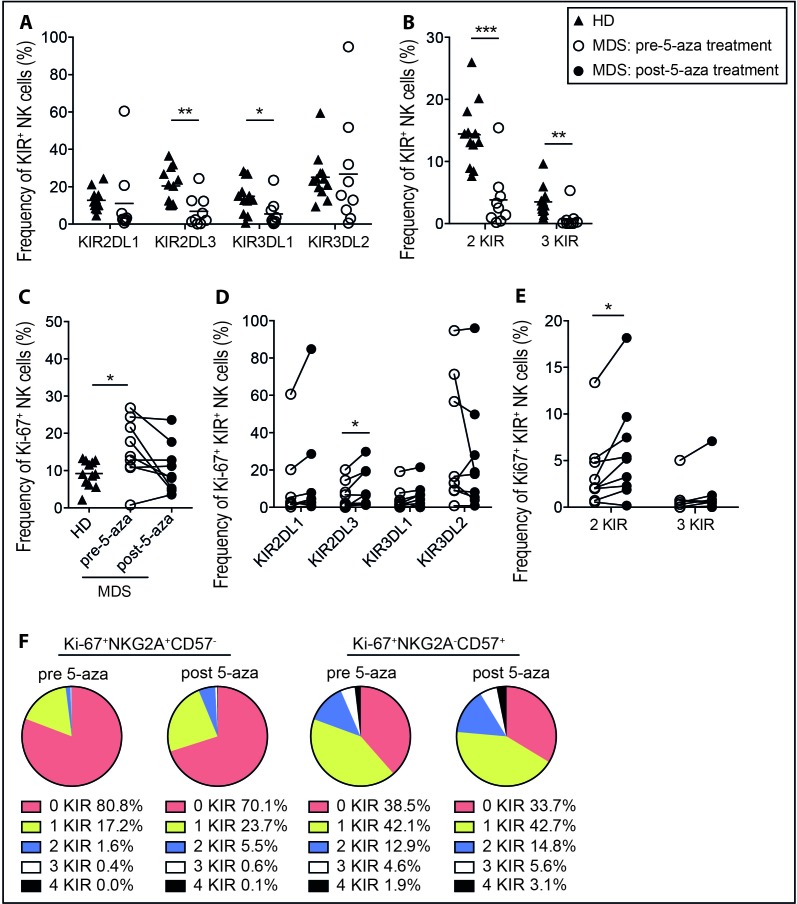
NK cell KIR repertoires of proliferating NK cells in high-risk MDS patients after 5-aza treatment PBMC were collected from healthy donors (HD), and MDS patients on day one pre-5-aza treatment and on day five post-5-aza treatment of cycle one. NK cells were analyzed directly *ex vivo* without culture. Shown for HD and pre-treatment patients in **A.** CD3^−^CD56^+^KIR2DL1^+^, -KIR2DL3^+^, -KIR3DL1^+^ and -KIR3DL2^+^ and in **B.** NK cells expressing multiple KIRs. Further, in **C.** the frequency of Ki-67^+^ NK cells in all groups, and in **D.** and **E.** the frequency of KIR2DL1^+^, -KIR2DL3^+^, -KIR3DL1^+^ and -KIR3DL2^+^ NK cells and cells expressing multiple KIRs within the Ki-67^+^ NK cell population in MDS patients, respectively. In **F.** the mean frequencies of Ki-67^+^NKG2A^+^CD57^−^ and Ki-67^+^NKG2A^−^CD57^+^ NK cell subsets expressing 0-4 KIRs in MDS patients. HD *n* = 12, MDS *n* = 9.

Next, we set out to test whether systemic 5-aza treatment had an influence on the NK cell repertoire in a cohort of high-risk MDS patients. To this end we first compared the baseline frequencies of NK cell subsets in healthy donors and patients. The overall frequency of NK cells and CD56^bright^ NK cells were similar in healthy controls and patients pre-treatment (Supplementary Figure 1A-1B). The distribution of NKG2A^+^CD57^−^ and NKG2A^−^CD57^+^ subsets varied greatly among patients with a tendency for higher frequencies of more immature subsets (Supplementary Figure 1C-1D) in line with previous findings [31]. Notably, however, the frequency of KIR2DL3^+^ and KIR3DL1^+^ NK cells, as well as NK cells expressing multiple KIRs, was significantly lower in MDS patients than in healthy donors (Figure 4A-4B). To follow dynamic changes during therapy, patients were samples at day one and day five in standard treatment regimes. On bulk NK cells the KIR repertoire was similar regardless of 5-aza, even when patients had undergone a total of 4 cycles of treatment (data not shown). However, as the *in vitro* data clearly showed that cell division was needed for 5-aza-induced KIR expression, we focused our analysis on the pool of cells expressing Ki-67, a surrogate marker for cells having undergone recent *in vivo* activation/proliferation [32]. Before treatment, patients displayed increased levels of Ki-67^+^ NK cells that returned to baseline on day five post-treatment (Figure 4C). In line with the *in vitro* proliferation data, more Ki-67^+^ cells were found within the less differentiated NKG2A^+^CD57^−^ NK cell subset than in the terminally differentiated NKG2A^−^CD57^+^ NK cell in healthy donors as well as patients (Supplementary Figure 1E). Notably, there was an increased frequency of Ki-67^+^ NK cells expressing KIR2DL3 after five days of 5-aza administration (Figure 4D). Furthermore, the frequency of cells expressing multiple KIRs was also increased (Figure 4E). When the Ki-67^+^ NK cells were stratified into NKG2A^+^CD57^−^ and NKG2A^−^CD57^+^ subsets, the effect of 5-aza was greater in the NKG2A^+^CD57^−^ subset (Figure 4F) concurrent with the *in vitro* data (Figure 3B). Thus, these data demonstrate that 5-aza treatment increases the total pool of KIR expressing NK cells, thereby partly restoring a mature NK repertoire in MDS patients.

### 5-aza does not accumulate in NK cells to give KIR expression upon induced proliferation

As 5-aza has a short half-life *in vivo* [28], cellular uptake and storage could play an important role in its mechanism of action. To address whether NK cells had been loaded with 5-aza *in vivo* but not yet undergone cell division and demethylation of KIR promoters, we monitored KIR repertoires in pre- and post 5-aza treatment patient samples after IL-2-induced proliferation *in vitro*. Proliferation rates of NK cells in patients were comparable to healthy donors and in most donors; systemic treatment with 5-aza lowered the ability of NK cells to proliferate in response to IL-2 (Supplementary Figure 2A). There was no effect on KIR expression regardless of the number of cell divisions during *in vitro* culture (Supplementary Figure 2B-2E), suggesting that NK cells had not accumulated intracellular stores of 5-aza.

Moreover, *in vitro* 5-aza addition to NK cells from MDS patients sampled pre-treatment induced KIR expression, although not to the extent observed in NK cells of healthy donors. In MDS patient samples, around 20% of NK cells that had divided three or more times in the presence of IL-2 and 5-aza still did not express any KIRs (Supplementary Figure 3A).

### 5-aza confers increased target cell responses of proliferating NK cells

**Figure 5:**
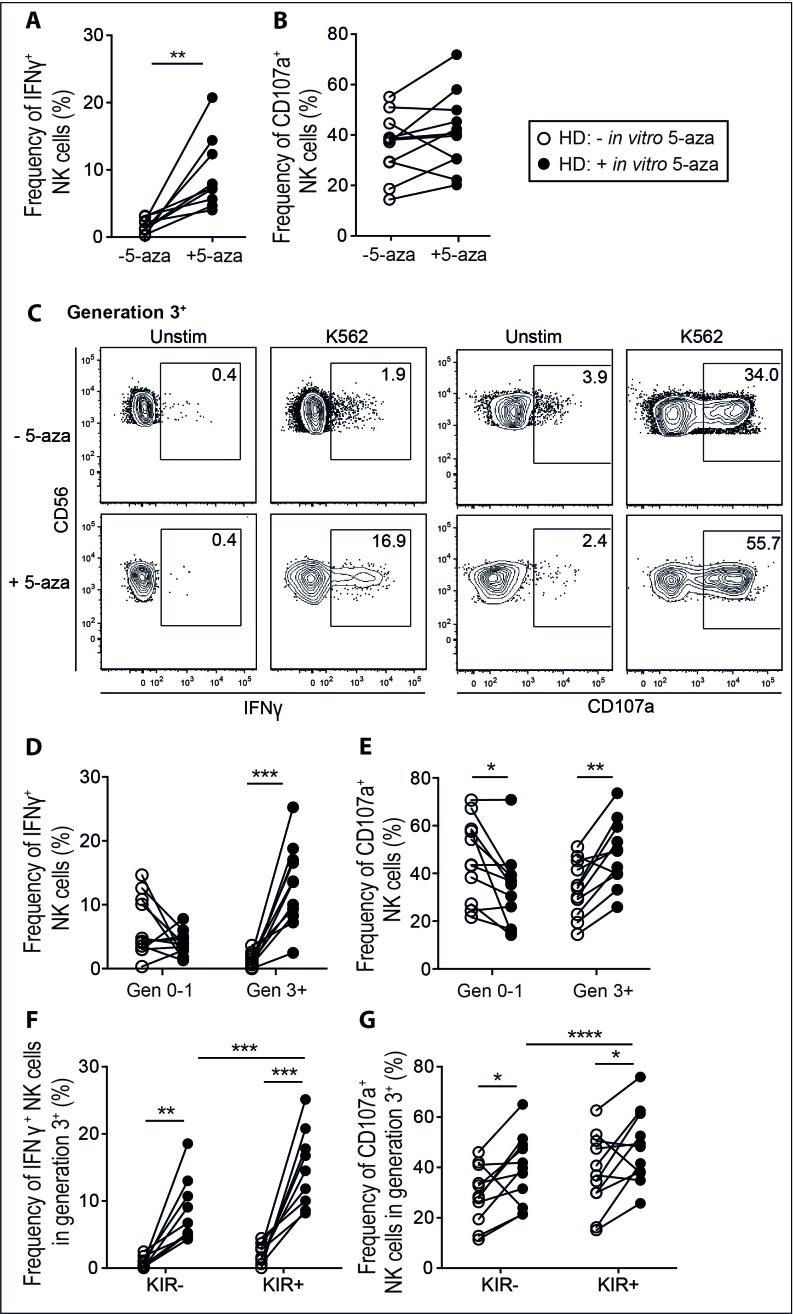
5-aza enhances function of proliferating NK cells towards tumor target cells NK cells were isolated from healthy donor PBMC and cultured in 500U/ml of IL-2 for six days with or without the addition of 5-aza for the first four consecutive days. Subsequently cells were cultured in IL-2 free medium for 48 h followed by a 4 h K562 degranulation assay. In **A.** and **B.** the frequency of responding cells in terms of IFN-γ production and CD107a mobilization within the total CD3^−^CD56^+^ NK cell population. In **C.** functional responses in generation 3^+^ NK cells from one representative donor, and quantified for generation 0-1 and 3^+^ for all donors in **D.** and **E.** In **F.** and **G.** stratification of generation 3^+^ NK cells into KIR^−/+^ subsets. HD *n* = 11.

Short-term exposure to demethylating agents has been reported to affect NK cell functionality [25, 33]. Here we monitored functional responses in discrete NK cell subsets in healthy donors following exposure to 5-aza *in vitro* and stratified the results based on cellular proliferation. Following the six-day IL-2 driven proliferation with or without 5-aza, we removed IL-2 for two days before subjecting the NK cells to a K562 tumor target cell assay. This rest period allowed for the induced effects of 5-aza to act independently of IL-2 induced hyper-responsiveness. As expected, KIR^+^ NK cells were more frequently CD107a^+^ and produced IFN-γ as compared to KIR^−^ cells following K562 stimulation (data not shown). 5-aza-treatment of NK cells led to significantly higher IFN-γ responses (Figure 5A and 5C), which was linked to proliferation (Figure 5D). Generation 3^+^ NK cells also had higher fractions of CD107a^+^ cells as compared to the same generation from untreated cultures (Figure 5E). Because of the variable frequencies of NK cells reaching generation 3, increased degranulation after 5-aza treatment was only visible at the bulk level in some donors (Figure 5B), correlating with killing of target cells as measured in a FACS-based killing assay (Supplementary Figure 4A-4B). Both KIR^−^ and KIR^+^ NK cells displayed enhanced responses to target cell stimulation following 5-aza treatment, although the KIR^+^ subset showed the largest increase in IFN-γ production (Figure 5F-5G). These results revealed that 5-aza had profound and replication-dependent effects on NK cell functionality, which were only partly linked to acquisition of inhibitory KIRs.

## DISCUSSION

MDS patients present with disturbed hematopoiesis in the myeloid lineage and defects in the number and subtypes of peripheral cells of multiple lymphoid lineages [31, 34-36]. MDS NK cells have impaired responses against tumor targets and immature subsets are overrepresented [31, 34, 36]. Here we show that 5-aza treatment increases expression of multiple KIRs in proliferating NK cells both *in vitro* and in MDS patients undergoing 5-aza treatment, and that 5-aza exposed proliferating NK cells display a higher functionality and may thus contribute to a beneficial anti-leukemic effect in 5-aza treated patients.

Previous reports demonstrated a profound effect on KIR expression in NK cells *in vitro* by the 5-aza related drug decitabine [21, 22]. However, decitabine has a largely different mode of action compared to 5-aza, involving incorporation only into DNA in contrast to the dual integration into both RNA and DNA of 5-aza [37]. Extending the results of Gao *et al.* [25] our high-resolution analysis of the NK cell KIR repertoire showed that low-dose 5-aza treatment of NK cells induced a significant expression of multiple inhibitory KIRs but only in proliferating cells. Thus, 5-aza was able to affect KIR promoter methylation despite the relatively lower degree of incorporation into DNA compared to decitabine. This is consistent with the need for only minor substitution of 5-aza for cytosine in DNA to inactivate more than 95% of DNMT1 [38]. We observed no preferential induction of specific KIRs or combinations of KIRs, suggesting a non-discriminate demethylation of the CpG islands adjacent to the KIR gene promotors, in line with the global demethylation by 5-aza observed in primary MDS CD34^+^ bone marrow cells [39].

Despite detailed knowledge on the effect of 5-aza on various immune cell subsets and cancer cell lines *in vitro*, little is known regarding 5-aza effects on the immune cell compartment *in vivo*. MDS patients had lower fractions of KIR-expressing NK cells pre-treatment and also tended to have lower frequencies of the most mature NKG2A^−^CD57^+^ NK cell subset. These data are consistent with those previously reported by Hejazi *et al.* [31], albeit they noted higher levels of KIR2DL3 in MDS patients. Interestingly, although we observed lower levels of all KIRs before therapy, the proliferating Ki-67^+^ fraction of NK cells displayed a selective increase in KIR2DL3, also manifested as higher frequencies of NK cells expressing multiple inhibitory KIRs. It was previously shown that the haplotype C1-specific KIR2DL2/3 is acquired earlier and with higher frequency than the C2-specific KIR2DL1 during NK cell differentiation [40-42] potentially explaining why the effect was most pronounced in this NK cell subset. Together these data indicate uptake of the drug into circulating cells and a demethylating effect of the drug *in vivo*. Both *in vitro* and *in vivo* when proliferating NK cells were stratified into subsets based on NKG2A and CD57 expression the effect of 5-aza was greater in the NKG2A^+^CD57^−^ subset, suggesting an overall skewing of the NK cell pool towards a more mature profile [30] under 5-aza influence. Notably, the *in vivo* effect of 5-aza was noted in the absence of IL-2 under steady-state. It is possible that the induction of KIR will be more pronounced if 5-aza is administrated together with immunomodulatory therapies or during the early reconstitution following stem cell transplantation.

When a standard dose of 5-aza is administered subcutaneously to patients, the highest levels measurable in plasma are similar, or slightly higher, to those that can induce demethylation *in vitro* [28, 43]. However, 5-aza is rapidly cleared from circulation with a half time in plasma of less than one hour [28] partly due to the action of the enzyme cytidine deaminase, which catalyzes the deamination of 5-aza, thereby destabilizing the drug and reducing the *in vivo* half-life [44]. Administration of 5-aza to MDS patients decreases the number of highly methylated loci but, surprisingly, the maximal effects on methylation levels appear one week after the end of drug administration [45] indicating that shifts in the NK cell KIR repertoire may be more noticeable at later time-points.

NK cells can contribute to the killing of MDS blast cells and disease progression may be associated with evasion of NK-cell mediated immune surveillance [46, 47]. Previous studies have demonstrated a strong association between reduced numbers and function of both peripheral and bone-marrow NK cells in MDS patients and disease severity [31, 34, 36, 47]. Here proliferating NK cells exposed to 5-aza *in vitro* exhibited considerably higher responses against K562 cells, suggesting that NK cells with an active uptake of 5-aza might mount improved responses against tumor targets. It is important to point out that this beneficial effect of 5-aza on NK cell cytotoxicity may only be relevant in conditions of intensified proliferation since resting NK cells are negatively affected even at low doses of 5-aza.

NK-cell education regulates cytotoxic responses against cellular targets and determines the functional potential of NK cells [15, 16, 30]. Surprisingly, the potentiating effect of 5-aza was evident in all cycling NK cells, irrespective of KIR status. This could possibly be a result of epigenetic remodeling of e.g. the IFN-γ locus, in similarity to what is observed during NK cell differentiation [48]. However, we observed no effect of 5-aza on the expression of granulysin, perforin or granzyme B or the activating receptors NKG2D or DNAM-1 (data not shown), indicating a lack of general upregulation of markers involved in NK cell activation/effector pathways. It is also possible that NK cells that had acquired KIR expression during cytokine-induced proliferation lost expression during the two days of IL-2 ‘rest’ and thus contributed to the response of the KIR- population. Indeed we noticed a drop in KIR expressing cells between day 6 and 8 in generation 3^+^ NK cells.

Other studies have shown that short-term treatment of resting NK cells with high doses of 5-aza lowers NK cell reactivity [25, 33]. Such replication-independent negative influence of 5-aza on NK cells that had not undergone cell division was also noted in our study despite the lower doses of 5-aza used. However, Schmiedel found that although initially impaired, IFN-γ production was recovered and even increased as compared to controls when NK cells were treated for 4 days with 5-aza under IL-2 stimulation [33] thereby supporting our results that proliferating cells with active incorporation of 5-aza acquire a heightened functionality. A recent study also demonstrated that decitabine treatment can depolarize Th2 cells to effectively secrete IFN-γ, signal via T-bet, and achieve demethylation of critical Th1 specific promoters [49]. The net effect of 5-aza on NK cell anti-tumor responses was recently investigated in B cell precursor acute lymphoblastic leukemia (BCP-ALL) bearing humanized mice. It was shown that 5-aza treatment reduced the tumor burden, which was attributed to improved NK cell functionality rather than direct effects of the drug on the tumor [50].

The prospect of engraftment of autologous or allogeneic NK cells for the treatment of hematopoietic malignancies has been highlighted in recent years [51]. The effects of 5-aza on the NK cell population may provide an avenue of experimental modification of KIR expression in this context. By selecting NK cells from donors with a given *KIR* genotype, and culturing these in the presence of 5-aza, it may be possible to increase KIR expression and thereby obtain cultures with high density of cells with enhanced function. Although 5-aza appears to induce expression of all KIRs, co-culture in the presence or absence of specific ligands may allow skewing of the KIR repertoires towards desired specificities [52]. Such efforts will require careful titration of the 5-aza doses, since the drug affects proliferative responses even at lower concentrations. 5-aza modulation of NK cells to yield specific subsets has been suggested for therapeutic applications for placental disorders associated with altered NK cell biology [53] and for pre-treatment of patients suffering from ALL [50].

In conclusion, these findings suggest that 5-aza remodeling of components of the immune system may act in concert with demethylating/cytotoxic effects on the malignant clones in MDS patients and warrants further investigations in NK cells of patients undergoing 5-aza treatment.

## MATERIALS AND METHODS

### Patients and healthy controls

The study was approved by the regional ethics committee [Stockholm, Sweden, approval number 2006/229-31/3] and was conducted in accordance with the Declaration of Helsinki. Patients (n=11) were recruited from the Hematology unit at the Karolinska University Hospital in Huddinge, Sweden. All patients gave their informed consent. Median age of the cohort was 74 years (range 45-83 years). According to the dosing schedule of the Nordic MDS guidelines, patients were administered 100 mg/m^2^ of 5-azacytidine (5-aza) subcutaneously in cycles consisting of one dose of 5-aza for five consecutive days followed by three weeks of rest. Blood samples were collected at day one (pre-treatment) and day five (post-treatment) of cycle one, and in some cases of cycle four. Healthy adult donor buffy coats were obtained from the blood bank at Karolinska University Hospital, Huddinge, Sweden. The frequencies of *KIR* A and B haplotypes were similar in healthy donors and patients (data not shown) as estimated based on expression patterns obtained with a 16-color KIR phenotyping panel, which resolves KIR2DL1, KIR2DS1, KIR2DL2/S2 expression [54, 55]. No *KIR* genotyping was performed.

### Cell processing

Peripheral blood mononuclear cells (PBMC) from healthy controls and patients were isolated by density gradient centrifugation (Ficoll-Hypaque; GE Healthcare) and NK cell phenotyping was performed directly *ex vivo* without culturing. The remaining PBMC were rested o.n. in culture medium (RPMI 1640 supplemented with 10% heat-inactivated human AB^+^ serum and 2 mM L-glutamine), followed by next-day negative isolation of NK cells (magnetic-activated cell sorting; Miltenyi Biotech). Isolated NK cells were labeled with 0.5 μM CellTrace Violet (Life Technologies), re-suspended in culture medium supplemented with 500 U/ml IL-2 (Peprotech) at a concentration of 1.5×10^6^ cells/ml and cultured at 37°C and 5% CO_2_ for six days. For *in vitro* 5-aza stimulation of healthy donor cultures or MDS patient samples, NK cells were treated with 1μM 5-azacytidine (Sigma) for the first 4 consecutive days of the culture. K562 cells were culture in RPMI 1640 supplemented with 10% FBS and 5 mM L-glutamine at 37°C and 5% CO_2_.

### Flow cytometry

For flow cytometry the following fluorochrome-conjugated specific antibodies were used: from Beckman Coulter; anti-CD3 PC5 (UHCT1), anti-CD56 ECD (NHK-1), anti-CD158a,h (KIR2DL1, KIR2DS1) PC7 (EB6B), anti-CD158b1/b2,j PC5.5 (GL183), custom made anti-NKG2A APC-AF750 (Z199). From BD Biosciences; anti-CD19 V500 (HIB19), anti-IgM eF650 (R6-60.2), anti-CD107a PE (H4A3), anti-Ki-67 PE (B56). From BioLegend; anti-IFN-γ BV785 (4S.B3), anti-KIR3DL1 AF700 (Dx9) and anti-CD56 BV421 (HCD56). From eBiosciences; purified anti-CD57 (TB01). From R&D systems; anti-KIR2DL1 APC (143211), anti-KIR2DL3 FITC (180701). From MabTech; anti-KIR3DL2 biotin (Dx31) with streptavidin QD605 (Invitrogen). From Life Technologies; CellTrace Violet™ Cell Proliferation Kit and LIVE/DEAD^®^ Fixable Aqua Dead Cell Stain Kit. From MBL: FITC conjugated caspase-3 substrate (FITC-VAD-FMK). Data were acquired in FACSDiva software on a BD LSR Fortessa equipped with a 488-nm laser, a 633-nm laser, a 405-nm laser, and a 562-nm laser. Acquired data were analyzed in FlowJo software (TreeStar, USA). Gating was performed on live CD19^−^CD3^−^CD56^+^ single cells within the lymphocyte gate or on CD56^−^ K562 cells within a larger forward and side scatter gate for killing assays.

### Functional assays

NK cell function was evaluated after the six-day IL-2-driven proliferation −/+ *in vitro* 5-aza (as described above) followed by a period of rest (48 h) in IL-2-free culture medium. NK cells were then mixed at a 1:1 ratio with K562 target cells and incubated in a V-bottom 96-well plate for 4 h at 37C and 5% CO_2_. For measurements of NK cell degranulation and intracellular cytokine production, Monensin (Golgi Stop, 1:1500, BD Biosciences), Brefeldin A (GolgiPlug, 1:1000, BD Biosciences) and anti-CD107a PE were added at the start of the 4 h co-incubation. At harvest, the cells were surface stained, followed by fixation and permeabilization (Fix/Perm kit, eBioscience) and subsequent intracellular staining for IFN-γ. For FACS-based evaluation of NK cell-mediated target cell killing, FITC conjugated caspase-3 substrate (FITC-VAD-FMK)(C3) was added at the start of the 4 h co-incubation. At harvest, cells were stained with LIVE/DEAD^®^ Fixable Aqua Dead Cell Stain (DCM) and CD56 BV421. The percentage of lysed target cells was determined as CD56^−^DCM^+^C3^+^ K562 cells. For all functional assays, values from unstimulated cultures were subtracted from stimulated cultures and subsequent statistical analysis was performed.

### Statistical analyses

Statistical differences were evaluated by Students t-test (non-paired or paired). All analyses were performed with GraphPad Software. Statistical significance was assumed when *p* < 0.05; **** indicates *p* < 0.0001, *** indicates *p* < 0.001, ** indicates *p* < 0.01, and * indicates *p* < 0.05.

## SUPPLEMENTARY MATERIAL FIGURES


