# Supplementary material for: Imprint of 5-azacytidine on the natural killer cell repertoire during systemic treatment for high-risk myelodysplastic syndrome
Source: Oncotarget. 2015 Oct 21;6(33):34178–90. doi: 10.18632/oncotarget.6213 (PMC4741444; doi:10.18632/oncotarget.6213)
Supplement: Supplementary file 1 [file oncotarget-06-34178-s001.pdf]

Imprint of 5-azacytidine on the natural killer cell repertoire during systemic treatment for high-risk myelodysplastic syndrome

Supplementary Material

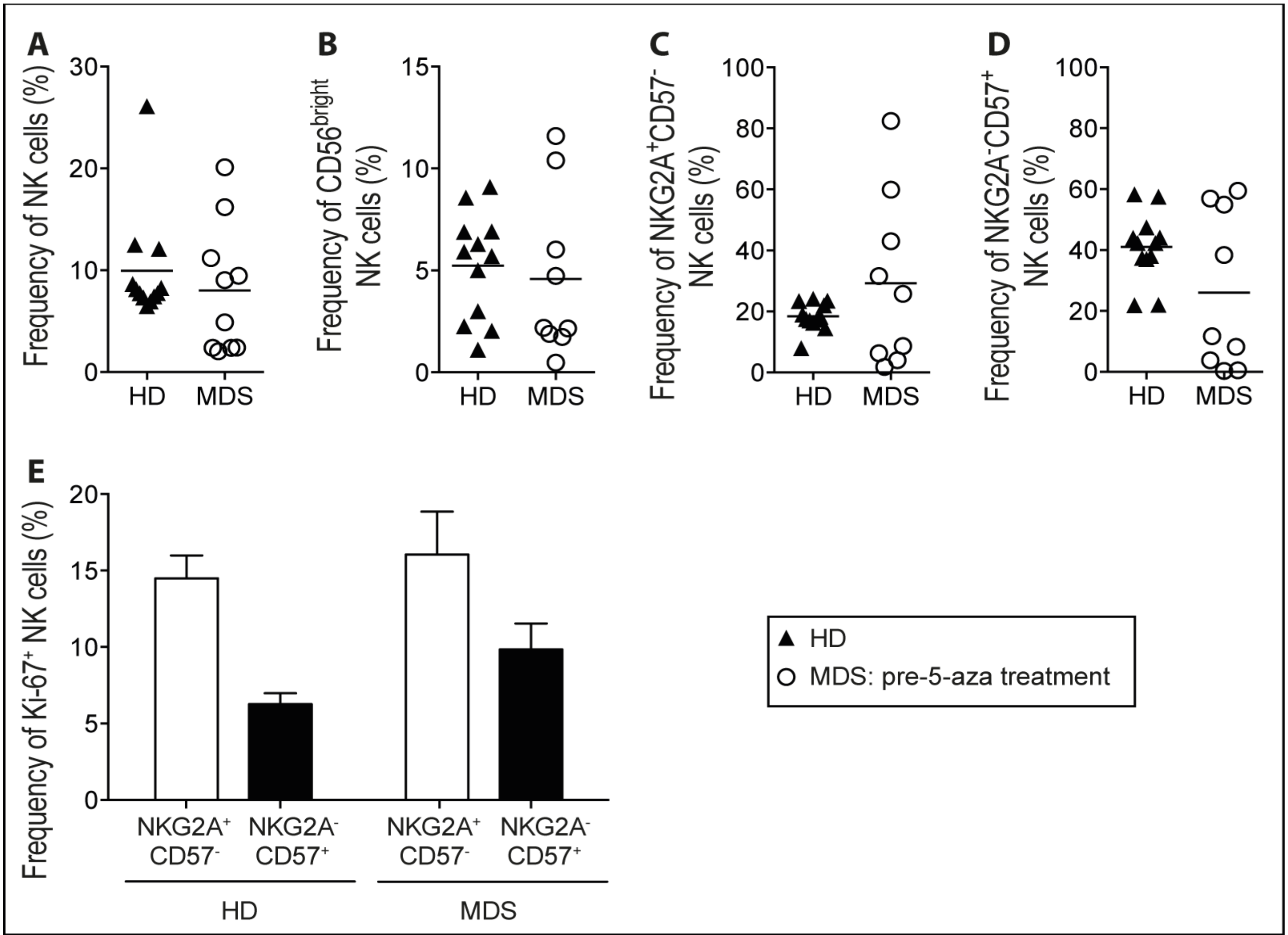

sFigure 1. Baseline frequencies of NK cell subsets in healthy donors and high-risk MDS patients. PBMC were collected from healthy donors (HD) and MDS patients on day one pre-5-aza treatment. NK cells were analyzed directly *ex vivo* without culture. Shown in **A.** the frequency of $CD3^-CD56^+$ NK cells, **B.** $CD56^{bright}$ NK cells, **C.** $NKG2A^+CD57^-$ NK cells, **D.** $NKG2A^-CD57^+$ NK cells and in **E.** Ki-67^+ NK cells within the $NKG2A^+CD57^-$ and $NKG2A^-CD57^+$ NK cell subsets. HD n=12, MDS n=9.

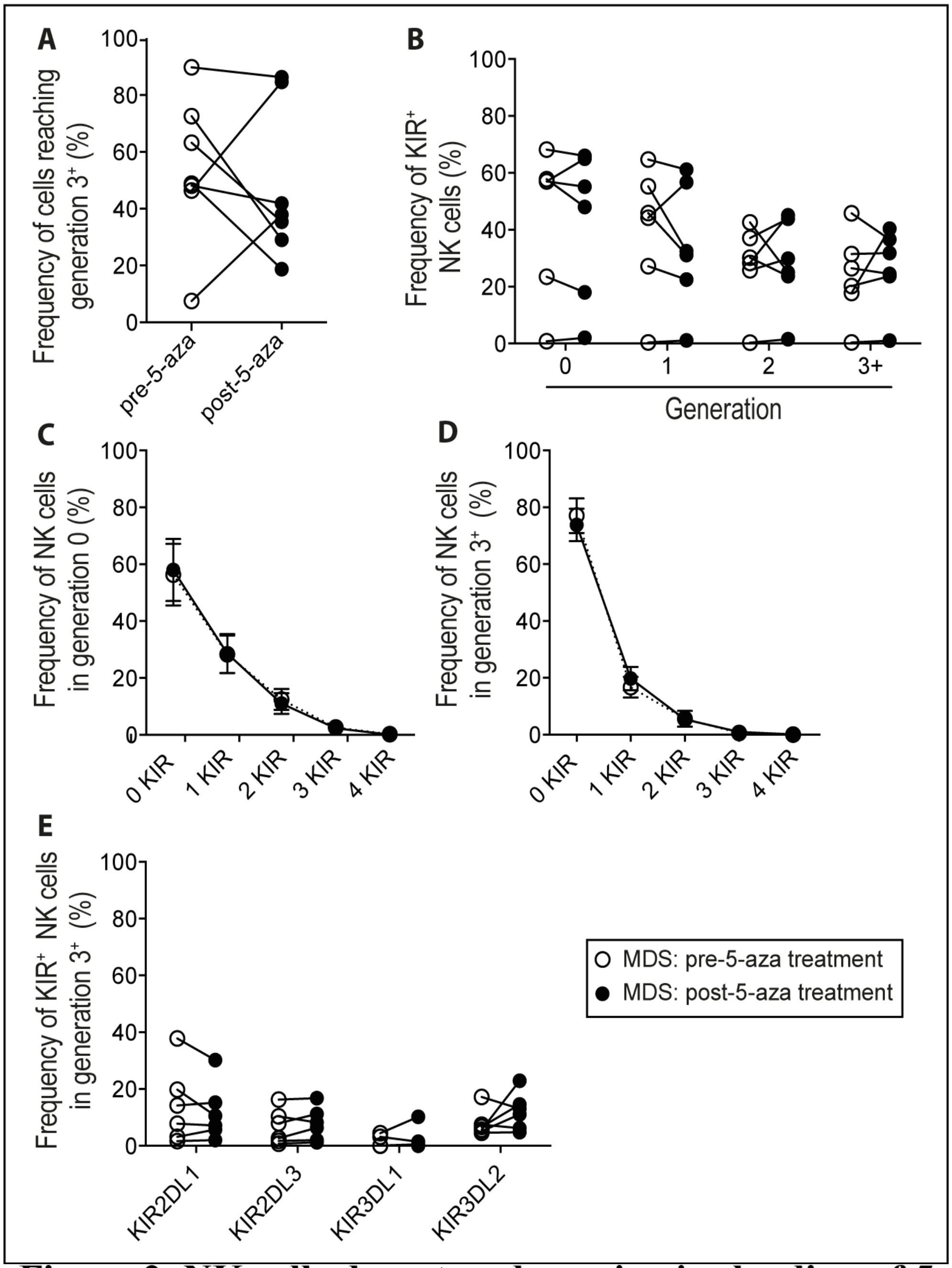

sFigure 2. NK cells do not undergo *in vivo* loading of 5-aza. PBMC were collected from MDS patients on day one pre-5-aza and day five post-5-aza treatment of cycle one. NK cells were isolated and cultured in 500U/ml of IL-2 for six days. In **A.** the frequency of CD3$^-$CD56$^+$ NK cells that had divided three or more times. In **B.** frequency of KIR$^+$ NK cells in each generation, **C.** the co-expression of multiple KIRs in generation 0 and in **D.** in generation 3$^+$. In **E.** the frequency of each investigated KIR in generation 3$^+$. MDS n=7.

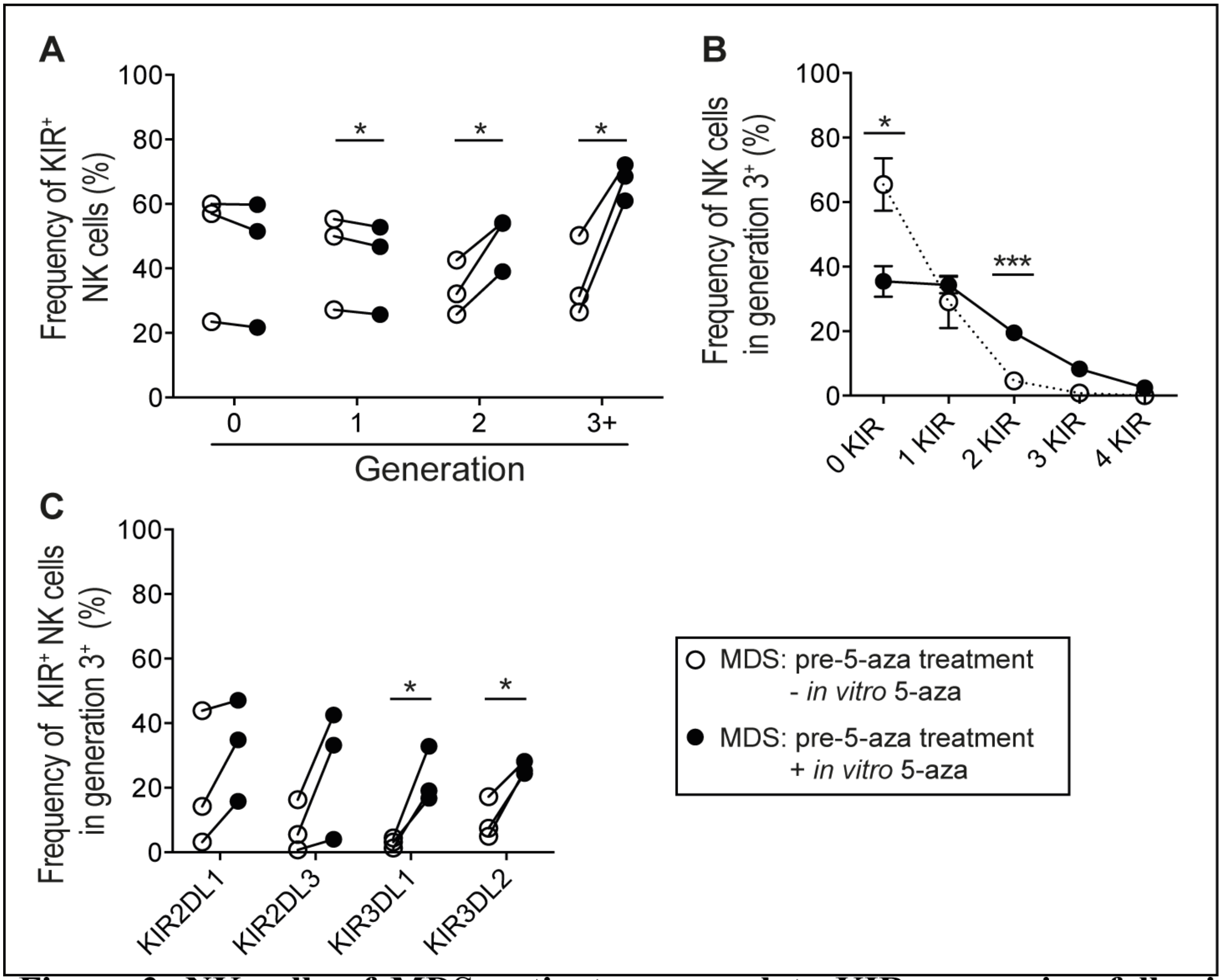

sFigure 3. NK cells of MDS patients up-regulate KIR expression following *in vitro* 5-aza addition. PBMC were collected from MDS patients on day one pre-5-aza treatment. NK cells were isolated and cultured in 500U/ml of IL-2 for six days with or without the addition of 5-aza for the first four consecutive days. Shown in **A.** the frequency of $CD3^{-}CD56^{+}KIR^{+}$ NK cells in each generation. For generation 3+ in **B.** the co-expression of multiple KIRs and in **C.** the frequency of each investigated KIR. MDS n=3.

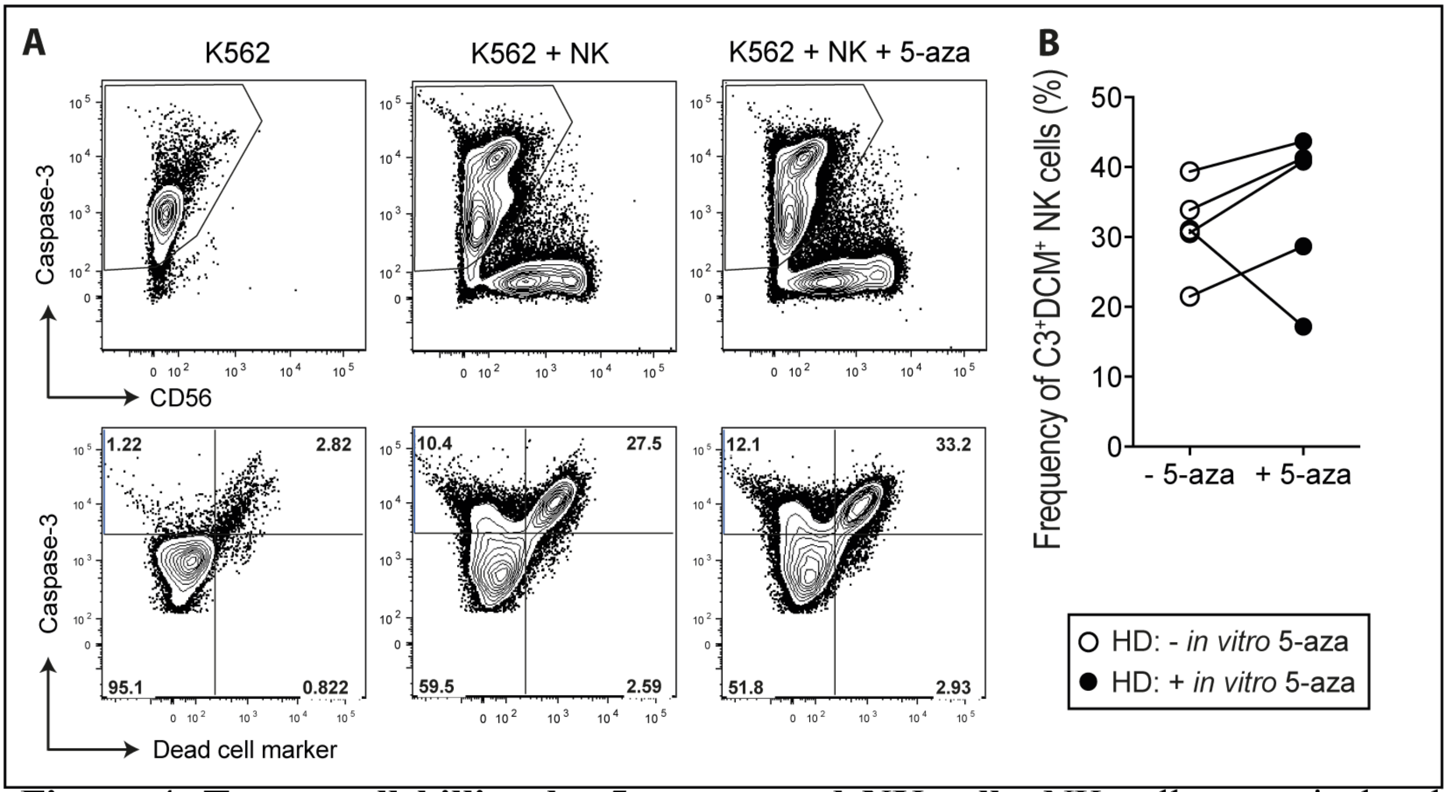

sFigure 4. Target cell killing by 5-aza treated NK cells. NK cells were isolated from healthy donor PBMC and cultured in 500U/ml of IL-2 for six days with or without the addition of 5-aza for the first four consecutive days. Subsequently cells were cultured in IL-2 free medium for 48 h followed by a 4 h K562 killing assay. The percentage of lysed K562 cells was defined as caspase-3 (C3) and dead cell marker (DCM) positive cells. In **A.** Gating strategy and target cell killing from one representative donor and in **B** quantified for all donors. HD n=5.
